# Alignment- and reference-free phylogenomics with colored de Bruijn graphs

**DOI:** 10.1186/s13015-020-00164-3

**Published:** 2020-04-07

**Authors:** Roland Wittler

**Affiliations:** 1 Genome Informatics, Faculty of Technology, Bielefeld University, Bielefeld, Germany; 2 Center for Biotechnology, Bielefeld University, Bielefeld, Germany; 3 Center for Biotechnology, Bielefeld Institute for Bioinformatics Infrastructure, Bielefeld University, Bielefeld, Germany

**Keywords:** Phylogenomics, Phylogenetics, Phylogenetic splits, Colored de Bruijn graphs

## Abstract

**Background:**

The increasing amount of available genome sequence data enables large-scale comparative studies. A common task is the inference of phylogenies—a challenging task if close reference sequences are not available, genome sequences are incompletely assembled, or the high number of genomes precludes multiple sequence alignment in reasonable time.

**Results:**

We present a new whole-genome based approach to infer phylogenies that is alignment- and reference-free. In contrast to other methods, it does not rely on pairwise comparisons to determine distances to infer edges in a tree. Instead, a colored de Bruijn graph is constructed, and information on common subsequences is extracted to infer phylogenetic splits.

**Conclusions:**

The introduced new methodology for large-scale phylogenomics shows high potential. Application to different datasets confirms robustness of the approach. A comparison to other state-of-the-art whole-genome based methods indicates comparable or higher accuracy and efficiency.

## Introduction

A common task in comparative genomics is the reconstruction of the evolutionary relationships of species or other taxonomic entities, their *phylogeny*. Today’s wealth of available genome data enables large-scale comparative studies, where phylogenetics is faced with the following problems: first, the sequencing procedure itself is becoming cheaper and faster, but finishing a genome sequence remains a laborious step. Thus, more and more genomes are published in an unfinished state, i.e., only assemblies (composed of contigs), or raw sequencing data (composed of read sequences) are available. Hence, traditional approaches for phylogenetic inference can often not be applied, because they are based on the identification and comparison of marker sequences, which relies on computing multiple alignments—an NP-hard task in theory, and in practice even heuristics are often too slow. Second, the low sequencing cost allow new large-scale studies of certain niches and/or aloof from model organisms, where reference sequences would be too distant or not available at all.

Whole-genome approaches that are usually alignment- and reference-free solve these problems, see e.g. [1–6]. However, the sheer number of genomes to be analyzed is still posing limits in large-scale scenarios as almost all whole-genome approaches are based on a pairwise comparison of some characteristics of the genomes (e.g. occurrences or frequencies of *k*-mers or other patterns) to define distances which are then used to reconstruct a tree (e.g. by using neighbor joining [7]). This means, for *n* genomes, $O(n^2)$ comparisons are performed in order to infer *O*(*n*) edges. To the best of our knowledge, only MultiSpaM [8] follows a different approach by sampling small, gap-free alignments involving *four* genomes each, which are used to infer a super tree on quartets. According to our experiments, this method is not suitable for large-scale settings, though (see Results).

Apart from computational issues, the actual objective of phylogenetic inference in terms of how to represent a phylogeny is not obvious in the first place. Taking only intra-genomic mutations into account, i.e., assuming a genome mutating independently of others, genomes would have unique lines of ancestors and their phylogeny would thus be a tree. Several reasons however conflict this simple tree model. Inter-genomic exchange of genomic segments such as crossover in diploid or polyploid organisms, lateral gene transfer in bacteria, or introgression in insects contradict the assumption of unique ancestry. Furthermore, incomplete, ambiguous, or even misleading information can hamper resolving a reliable phylogenetic tree.

Here, we propose a new methodology that is whole-genome based, alignment- and reference-free, and does not rely on a pairwise comparison of the genomes or their characteristics. An implementation called SANS (“Symmetric Alignment-free phylogeNomic Splits”) is available online [9]. The *k*-mers of all genomic sequences (assemblies or reads) are stored in a colored de Bruijn graph, which is then traversed to extract phylogenetic signals. The reconstructed phylogenies are not restricted to trees. Instead, the generalized model of *phylogenetic splits*  [10] is used to infer phylogenetic networks that can indicate a tree structure and also point to ambiguity in the reconstruction.

In the following "Background" section, we will first introduce two building blocks of our approach, *splits* and *colored de Bruijn graphs*. Then, we will describe and motivate our method in the "Method" section. After an evaluation on several real data sets in the "Results" section, we will give a brief summary and an outlook in the "Conclusions" section.

A preliminary version of this study has been published at the *Workshop on Algorithms in Bioinformatics* (WABI) 2019 [11].

## Background

Before presenting our method in the "Method" section, we will introduce two basic concepts it builds upon. Firstly, as motivated above, our phylogenies will be represented by sets of *splits*, a generalization of trees. Secondly, to extract phylogenetic signals from the given genomes in the first place, they are stored in a *colored de Bruijn graph*.

### Phylogenetic splits

In the following, we briefly recapitulate some notions and statements from the split decomposition theory introduced by Bandelt and Dress [10], and put them into context.

#### **Definition 1**

*(Unordered split)* Given a nonempty set *O*, if for two proper subsets $A,B\subseteq O$, both $A\cap B=\emptyset$ and $A\cup B=O$, then the unordered pair $\{A,B\}$ is a *bipartition* or *(unordered) split* of *O*. If either *A* or *B* is of cardinality one, a split is called *trivial*.

We extend the above commonly used terminology of (unordered) splits to *ordered splits*—a central concept in our approach.

#### **Definition 2**

*(Ordered split)* If $\{A,B\}$ is an unordered split of *O*, the ordered pairs (*A*, *B*) and (*B*, *A*) are *ordered splits*. (*B*, *A*) is called the *inverse (split)* of (*A*, *B*) and *vice versa*.

Note that one unordered split $\{A,B\}=\{B,A\}$ corresponds to two ordered splits $(A,B) \ne (B,A)$. Our method will first infer ordered splits and their inverse, which will then be combined to form unordered splits. If clear from the context, we may denote an ordered split (*A*, *B*) by simply *A*.

A set of splits $\mathcal {S}$ may be supplemented with weights $w:\mathcal {S}\longrightarrow \mathbb {R}$, e.g., in [10], splits are weighted by a so-called *isolation index*. Strong relations between metrics and sets of weighted unordered splits have been shown. In particular, one can canonically decompose any metric distance *d* into a set of weighted splits $\mathcal {S}_d$ that is *weakly compatible* in the following sense.

#### **Definition 3**

*(Weak compatibility* [10]*)* A set of unordered splits $$\mathcal {S}$$ on *O* is *weakly compatible* if for any three splits $$\{A_1,B_1\}$$, $$\{A_2,B_2\}$$, $$\{A_3,B_3\}$$$$\in \mathcal {S}$$, there are no elements $$a, a_1, a_2, a_3 \in O$$ with $$\{a,a_1, a_2, a_3\}\cap A_i = \{a,a_i\}$$ for $$i=1,2,3$$.

Then, $d(a,b) = \sum _{\{A,B\} \in \mathcal {S}_d}{w(\{A,B\}) \, \delta _{A}(a,b)} + d_0$ where $\delta _{A}(a,b):=1$ if either *a* or *b* in *A*, but not both, and $\delta _{A}(a,b):=0$ otherwise, i.e., the weights of all splits separating *a* from *b* are accumulated, and where $d_0$ is a so-called *split prime* residue that cannot be decomposed further.

As a peculiarity of our approach is being *not* distance-based, we mention the above relation of weakly compatible splits and distances only for the sake of completeness. We will make use of the above property to filter a general set of splits such that it can be displayed as a—in most cases planar—network.

A metric *d* is a tree metric (also called *additive*), if and only if there is a set of splits $\mathcal {S}_d$ with $d(a,b) = \sum _{\{A,B\} \in \mathcal {S}_d}{w(\{A,B\}) \, \delta _{A}(a,b)}$ that is *compatible* in the following sense.

#### **Definition 4**

*(Compatibility* [10]*)* A set of unordered splits $\mathcal {S}$ on *O* is *compatible* if for any two splits $\{A,B\}$ and $\{A',B'\}$, one of the four intersections $A\cap A'$, $A\cap B'$, $B\cap A'$, and $B\cap B'$ is empty.

We will make use of the implied one to one correspondence of edges in a tree and compatible splits: an edge of length *w* whose removal separates a tree into two trees with leaf sets *A* and *B*, respectively, corresponds to a split $\{A,B\}$ of weight *w*.

### Colored de Bruijn graphs

A *string* *s* is a sequence of characters over a finite, non-empty set, called *alphabet*. Its *length* is denoted by |*s*|, the character at position *i* by *s*[*i*], and the substring from position *i* through *j* by *s*[*i*..*j*]. A string of length *k* is called *k-mer*.

We consider a *genome* as a set of strings over the DNA alphabet $\{A,C,G,T\}$. The *reverse complement* of a string *s* is $\overline{s}:=\overline{s[|s|]}\cdots \overline{s[1]}$, where $\overline{A}:=T, \overline{C}:=G, \overline{G}:=C, \overline{T}:=A$.

An abstract data structure that is often used to efficiently store and process a collection of genomes is the *colored de Bruijn graph (C-DBG)* [12]. It is a node-labeled graph (*V*, *E*, *col*) where each vertex $v \in V$ represents a *k*-mer associated with a set of colors *col*(*v*) representing the set of genomes the *k*-mer occurs in. A directed edge from *v* to $v'$ exists if and only if for the corresponding *k*-mers *x* and $x'$, respectively, $x[2..k] = x'[1..k-1]$. We call a path $p=v_1,\ldots ,v_l$ of length $|p|=l$ in a C-DBG *non-branching* if all contained vertices have an in- and outdegree of one with the possible exception of $v_1$ having an arbitrary indegree and $v_l$ having an arbitrary outdegree, and it has the same set of colors assigned to all its vertices. A maximal non-branching path is a *unitig*. In a *compacted C-DBG*, all unitigs are merged into single vertices.

In practice, since a genomic sequence can be read in both directions, and the actual direction of a given sequence is usually unknown, a string and its reverse complement are assumed equivalent. Thus, in many C-DBG implementations, both a *k*-mer and its reverse complement are represented by the same vertex. In the following, we will assume this being internally handled by the data structure.

## Method

The basic idea of our new approach is that a sequence which is contained as substring in a subset *A* of all genomes *G* but not contained in any of the other genomes is interpreted as a signal that *A* should be separated from $G\backslash A$ in the phylogeny. The more of those sequences exist and the longer they are, the stronger is the signal for separation.

To efficiently extract common sequences, we first construct a C-DBG of all given genomes. Then, we collect all separation signals as ordered splits, where any unitig *u* contributes |*u*| to the weight of an ordered split *col*(*u*). Since both an ordered split (*A*, *B*) and its inverse (*B*, *A*) indicate that *A* and *B* should be separated in the phylogeny, we combine them to one unordered split $\{A,B\}$ with an overall weight that is a combination of the individual weights. The individual steps will be explained in more detail next.

*C-DBG*. Among several available implementations of C-DBGs (e.g. [12–15]), we decided to use Bifrost [16] for the following reasons: it is easy to install and use; it is efficiently implemented; it can process full genome sequences, assemblies, read data or even combinations of these; for read data as input, it offers some basic assembly-like filtering of *k*-mers; and it realizes a compacted C-DBG and provides a C++ API such that a traversal of the unitigs could be easily and efficiently implemented—only unitigs with heterogeneous color sets had to be split, because colors are not considered during compaction.

Like other implementations of DBGs on the DNA alphabet, Bifrost saves space by not storing edges explicitly—with the trade-off of having to determine neighboring vertices by querying the graph for all possible preceding and succeeding *k*-mers. Since we do not make use of the topology of the C-DBG, this common design decision accommodates our needs.

**Fig. 1:**
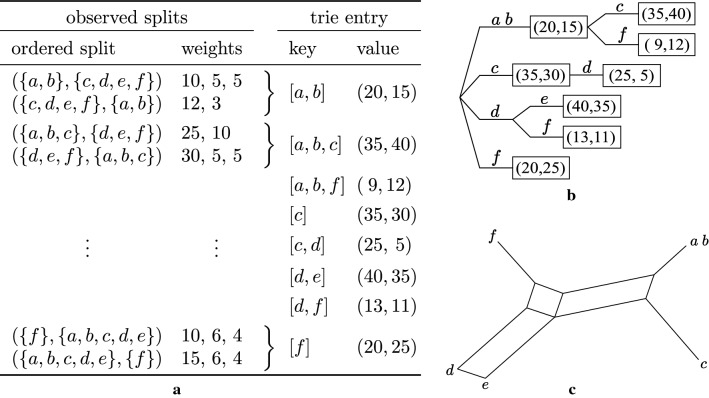
Toy example for accumulating weights in a trie. The set of genomes $\{a,b,c,d,e,f\}$ is considered as ordered list $G=[a,b,c,d,e,f]$. **a** A table listing ordered splits extracted from a C-DBG and the corresponding representation as key and value to be stored in a trie. An ordered split can be observed several times with different weights, which are accumulated. A split and its inverse are represented in the trie by one key and a pair of weights. **b** Trie representing the splits. Keys correspond to concatenated edge lables along paths from the root to a labeled vertex. Values are shown in boxes. **c** Visualization of weakly compatible subset of splits. E.g., split $(\{a,b,f\},\{c,d,e\})$ of total weight $\sqrt{9\cdot 12}\approx 10.4$ is visualized as three parallel lines. Split $(\{c,d\},\{a,b,e,f\})$ has the lowest total weight of $\sqrt{25\cdot 5}\approx 10.2$, is the only split that is not weakly compatible to higher weighting splits and thus not contained in the network

*Accumulating split weights*. Because different splits often have many genomes in common, we use a trie data structure to store a split (as key) as path from the root to a vertex, along with its weight (as value) assigned to that vertex. We represent the set of genomes *G* as a list with some fixed order, and any subset of *G* as a sublist, i.e., with the same relative order. For a split (*A*, *B*) and its inverse (*B*, *A*), we take as key the shorter of *A* and *B*, breaking ties by selecting that split containing *G*[0], and as value the pair of weights $(w,w')$, where *w* is the accumulated weight of the key, and $w'$ the accumulated weight of its inverse. When the trie is accessed for a key the first time, the value is initialized with (0, 0). For further observations of the same split, the corresponding entry is increased. Figure 1 shows an example.

The overall method SANS is shown in Algorithm 1, the very last step of which will be motivated in the following. 
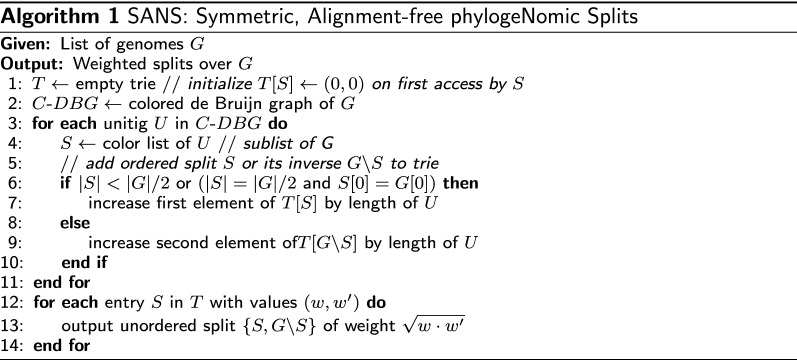


*Combining splits and their inverses*. To combine an ordered split (*A*, *B*) of weight $w_A$ and its inverse (*B*, *A*) of weight $w_B$, a naive argument would be: both indicate the same separation, so they should be taken into account equivalently, and thus take the sum $w_A + w_B$ or arithmetic mean $(w_A + w_B)/2$. However, in our evaluation, this weighting scheme often assigned higher weight to wrong splits than to correct splits (compared to reliable reference trees; exemplified in Sect. "Drosophila"). Instead, we revert the above argument: consider a mutation on a (true) phylogenetic branch separating the set of genomes into subgroups *A* and *B*. The corresponding two variants of the affected segment will induce two unitigs with color sets *A* and *B*, respectively. Under the infinite sites assumption, these unitigs would not be affected by other events. So, each mutation on a branch in the phylogeny contributes to *both* splits (*A*, *B*) and (*B*, *A*). We hence take the geometric mean $\sqrt{w_A \cdot w_B}$ such that in case of asymmetric splits, the lower weight diminishes the total weight, and only symmetric splits receive a high overall weight.

**Fig. 2:**
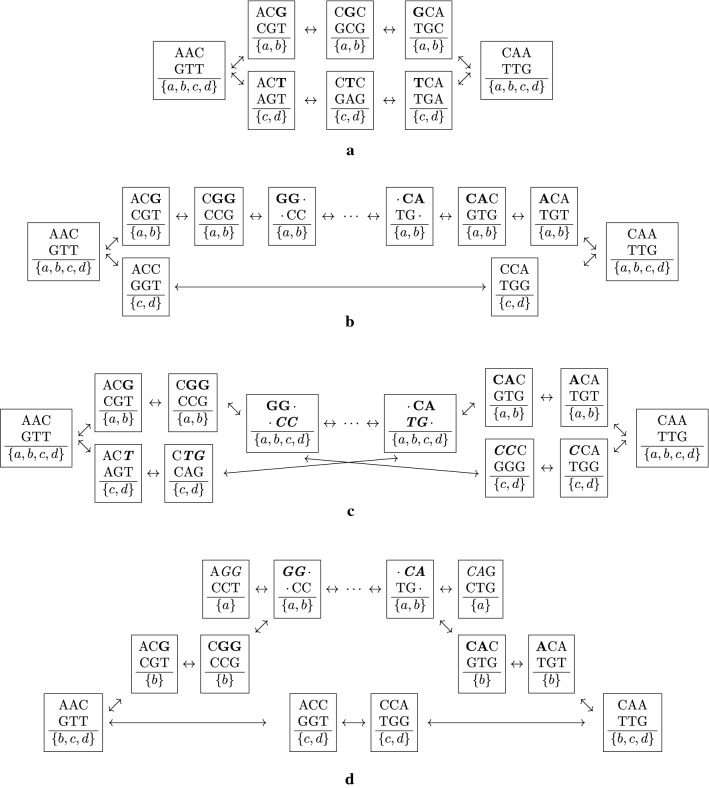
Toy examples for different mutations to illustrate their effect on a C-DBG. Consider four genomes *a*, *b*, *c* and *d* and *k*-mer length $k=3$. Each vertex of the C-DBG is labeled with both its *k*-mer and the reverse complement (in arbitrary order), as well as its color set. Due to the small value of *k*, the C-DBG contains edges corresponding to pairs of overlapping *k*-mers that are not contained in the given strings. For the purpose of clarity, these are not drawn. Mutations are highlighted in bold and/or italics. **a** Single nucleotide variation in genomes $a=b=\;$AAC**G**CAA and $c=d=\;$AAC**T**CAA. The induced ordered split $\{a,b\}$ and its inverse $\{c,d\}$ of weight $k=3$ each yield a corresponding unordered split $\left\{ \{a,b\},\{c,d\}\right\}$ of weight $\sqrt{k\,k}=k=3$. **b** Insertion/deletion of length $l=4$ (or longer, indicated by dots) in genomes $a=b=\;$AAC**GG**$\,\cdots$**CA**CAA and $c=d=\;$AACCAA. The induced ordered split $\{a,b\}$ of weight $l+k-1 = l+2$ and its inverse $\{c,d\}$ of constant weight $k-1 = 2$ yield a corresponding unordered split $\left\{ \{a,b\},\{c,d\}\right\}$ of weight $\sqrt{(l+k-1)\,(k-1)}=\sqrt{2(l+2)}$. **c** Inversion of length $l=4$ (or longer, indicated by dots) between genomes $a=b=\;$AAC**GG**$\,\cdots$**CA**CAA and $c=d=\;$AAC**TG**$\,\cdots$**CC**CAA. The induced ordered split $\{a,b\}$ and its inverse $\{c,d\}$ of constant weight $2(k-1) = 4$ each yield a corresponding unordered split $\left\{ \{a,b\},\{c,d\}\right\}$ of constant weight $\sqrt{2(k-1)\,2(k-1)}=2(k-1)=4$. **d** Lateral gene transfer of length $l=4$ (or longer, indicated by dots) from genome $a=\;$A*GG*$\,\cdots$*CA*G to $b=\;$AAC**GG**$\,\cdots$**CA**CAA but not to $c=d=\;$AACCAA. Apart from mutation-independent splits for the boundaries, and the trivial split $\{b\}$ (without its inverse), the split $\{a,b\}$ of weight $l-k+1=l-2$ and its inverse $\{c,d\}$ of constant length $k-1=2$ are induced, yielding a corresponding unordered split $\left\{ \{a,b\},\{c,d\}\right\}$ of weight $\sqrt{(l-k+1)\,(k-1)}=\sqrt{2(l-2)}$

Considering different scenarios that would affect the observation of common substrings in the C-DBG, some of which are illustrated in Fig. 2, we observe beneficial behavior of the weighting scheme in almost all cases:

A *single nucleotide variation* would cause a bubble in the C-DBG composed of two unitigs of similar length *k* each—a symmetric scenario in accordance with the above weighting scheme. Both an *insertion or deletion* of length *l* would cause an asymmetric bubble and thus asymmetric weights $k-1$ and $l+k-1$. Here, the geometric mean has the positive effect to weaken the impact of the length of the event on the overall split weight. E.g., the total weight for *x* deletions of length *l* would increase linearly with *x* whereas those for one deletion of length $x\cdot l$ would increase with $\sqrt{x}$. For both a *transposition or inversion* of arbitrary length, the color set of the segment itself remains the same, and only those *k*-mers spanning the breakpoint regions would be affected, inducing symmetric bubbles in accordance with the weighting scheme. *Lateral gene transfer* is challenging phylogenetic reconstruction, because a subsequence of length *l* that is contained in both the group *A* containing the donor genome as well as the target genome *b* from the other genomes $B:= G \backslash A$ can easily be misinterpreted as a signal to separate $A\cup \{b\}$ from the remainder $B\backslash \{b\}$ instead of separating *A* from *B*, where the strength of this erroneous signal grows with *l*. Our approach will be affected only little: on the one hand, the unitig corresponding to the copied subsequence has color set $A\cup \{b\}$ and thus contributes to an ordered split $(A\cup \{b\},B\backslash \{b\})$ of weight $l-k+1$. On the other hand, because the transfer does not remove any subsequence in the donor sequence, only those *kk*-mers spanning the breakpoint region will be affected, inducing a unitig with color set $B\backslash \{b\}$ whose length is independent of *l*. *Missing or additional data* may arise from genomic segments that are difficult to sequence or assemble and might thus be missing in some assemblies, due to the usage of different sequencing protocols, assembly tools, or filter criteria, or simply because some input files contain plasmid or mitochondrial sequences and others do not. This does not affect our approach, because additional sequence induces unitigs and thus an ordered split, but the absence of sequence does not induce any split, not even due to breakpoint regions, because in such cases usually whole reads, contigs or chromosomes are involved. Thus, the weight of the additional ordered split would be multiplied by zero for the absent split, resulting in a total weight of zero. *Copy number changes* can only be detected if the change is from one to two or *vice versa*, adding or removing *k*-mers spanning the juncture of the two copies. Beyond that, because the *k*-mer counts are not captured, our approach is not sensible for copy number changes.

In practice, the structure of a C-DBG is much more complex than the simplified picture we draw above. Nevertheless, using the geometric mean yields high accuracy of the approach compared to other methods.

*Postprocessing*. Even though the geometric mean filters out many asymmetric splits, the total number of positively weighted splits can be many-fold higher than $2n-3$, the number of edges in a fully resolved tree for *n* genomes. Unfortunately, the observed distribution of split weights did not indicate any obvious threshold to separate high-weighted splits from low-weighted noise. Nevertheless, a rough cutoff can safely be applied by keeping only the *t* highest weighting splits, e.g., in our evaluation $t=10\,n$ has been used for all datasets. Additionally, we evaluated two filtering approaches: *greedy weakly*, i.e., greedily approximating a maximum weight subset that is weakly compatible and can thus be displayed as a network, and *greedy tree*, i.e., greedily approximating a maximum weight subset that is compatible and thus corresponds to a tree. To this end, we used the corresponding options of the software tool SplitsTree [17, 18]. As we will demonstrate in Sect. "Results", in particular the tree filter proved to be very effective in practice.

*Run time complexity*. Consider *n* genomes of length *O*(*m*) each. In Bifrost, the compacted C-DBG is built by indexing a *k*-mer by its *minimizer*, i.e., a substring with the smallest hash value among all substrings of length *g* in a *k*-mer. According to the developers of Bifrost (personal communication), inserting a *k*-mer and its color takes $O(4^{(k-g)} log(n))$ time in the worst case. In practice, however, each of the $O(m\,n)$*k*-mers can be inserted in $O(\log (n))$ time, and hence, building the complete C-DBG takes $O(m\,n\log (n))$ time. While iterating over all positions in the graph, we verify whether a unitig has to be split due to a change in the color set. Because each of the *n* genomes adds *O*(*m*) color assignments to the graph, we have to do $O(m\,n)$ color comparisons in total, which does not increase the overall complexity.

Each genome contributes to at most *O*(*m*) ordered splits. So the sum of the cardinality of all ordered splits, i.e., the total length of all splits in Algorithm 1, is $O(m\,n)$. Hence, the insertion and lookups of all *S* in trie *T* takes |*S*| time each and $O(m\,n)$ in total, and the number of vertices of *T*, i.e., the final number of unordered splits, is in $O(m\,n)$, too. For ease of postprocessing, splits are ordered by decreasing weight, increasing the run time for split extraction to $O(m\,n\log (m\,n))$, or $O(m\,n\log (n))$ to output only the *t*, $t\in O(n)$, highest weighting splits, respectively.

## Results

In this section, we present several use cases in order to exemplify robustness and different other characteristics of our approach SANS. We compare to the following other whole-genome based reconstruction tools.

MultiSpaM [8] samples a constant, high number of small, gap-free alignments of four genomes. The implied quartet topologies are combined to an overall tree topology. To the best of our knowledge, all other tools are distance-based and rely on pairwise comparisons. Interestingly, although all methods are based on lengths or numbers of common subsequences or patterns, their results differ considerably from those of SANS. Co-phylog [4] analyzes each genome in terms of certain patterns *(C-grams, O-grams)* and compares their characteristics *(context)*. In andi [2], enhanced suffix arrays are used to detect pairs of maximal unique matches that are used to anchor ungapped local alignments, based on which pairwise distances are computed. CVTree3 [6] corrects *k*-, $(k\!-\!1)$-, and $(k\!-\!2)$-mer counts by subtracting random background of neutral mutations using a $(k\!-\!2)$-th Markov assumption. In FSWM [3], matches of patterns including match and *don’t-care* position are scored and filtered to estimate evolutionary distances.

Unless stated otherwise, a *k*-mer length of 31 (Bifrost default) has been used for constructing the C-DBG for SANS. All tools have been run on a single 2 GHz processor and times are given in CPU hours (user time). Accuracy has been measured in terms of topological Robinson-Foulds distance, i.e., a predicted edge (split) is correct if and only if the reference tree contains an edge that separates the same two sets of leaves. As *recall*, we count the number of correct edges (splits) divided by the total number of edges in the reference, and as *precision*, we count the number of correct edges (splits) divided by the total number of predicted edges (splits).

### *Drosophila*

This dataset comprises assemblies from 12 species of the genus *drosophila* obtained from the database FlyBase (latest release before Feb. 2019 of all-chromosome-files each) [19]. As reference, we consider the commonly accepted phylogeny published by the FlyBase consortium [20, Figure 2] also shown on the database website.

**Fig. 3:**
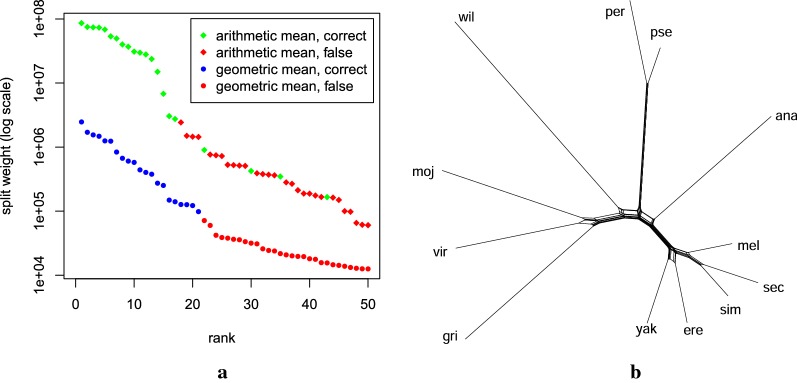
Reconstructed phylogenetic splits on the Drosophila dataset [19]. **a** Comparison of accuracy for using arithmetic or geometric mean for combining weights of splits and their inverse each. Splits have been sorted by the combined weight and the 50 highest weighting splits are shown. Color indicates whether a split agrees with the reference [19]. **b** Visualization of greedily extracted weakly compatible subset of splits using SplitsTree [17, 18]. As by default, geometric mean has been used for combining weights of splits and their inverse each

Although being “simple” in the sense that it contains only a small number of genomes, its analysis exemplifies the following aspects: (i) The effectiveness of our method for medium sized input files: for a total of more than 2 161 Mbp (180 Mbp on average), SANS inferred the correct tree within 168 min and using up to 25 GB of memory. We ran CVTree3 with various values of *k*. In the best cases ($k=12$ and 13), 7 of 9 internal edges have been inferred correctly taking 95 and 162 min, and up to 26 and 87 GB of memory, respectively. (For $k=11$, only 4 internal edges were correct, and for $k>13$, the computation ran out of memory.) Both Co-phylog and FSWM did not finish within 48 hours, and both MultiSpaM and andi could not process this dataset successfully. (ii) As can be seen in Fig. 3a, combining splits and their inverse using the geometric instead of the arithmetic mean strengthens the tendency of correct splits having a high weight. (iii) Even though the reconstruction shown in Fig. 3b contains 45 splits—in comparison to 21 edges in a binary tree—, the visualization is close to a tree structure.

*Association of splits and sequences* Here, we want to highlight a distinctive feature of our methodology. In contrast to distance based approaches, there is a one-to-one correspondence of a phylogenetic split to the conserved sequences it is derived from. This provides the opportunity to trace the cause for a phylogenetic signal back to the sequence level. Splits of particular interest might be, e.g., those separating pathogens from non-pathogens, splits contradicting each other, or—as in this case of *Drosophila* with a well-accepted reference at hand—splits contradicting the species phylogeny.

As a proof of concept, we analyzed a predicted split from the weakly compatible subset of splits shown in Fig. 3b which disagrees with the reference phylogeny. We selected the highest weighting split which separates at least 25 percent of the genomes: *mel, sec, sim, ere* separating from the rest. This split was supported by 290 8641 *k*-mers contained in the four genomes but not in the others, and 456 *k*-mers contained in the other genomes but not in the four, resulting in a total weight of approximately 36 419. In comparison, the weakest true positive split has a weight of approximately 98 290. Searching the sequence of the longest unitig involved in the selected false positive split in the standard database *Nucleotide collection (nr/nt)* using NCBI blastx with default parameters yields seven matches, the four highest with E-values ranging from $3\cdot 10^{-15}$ to $2\cdot 10^{-12}$ being:

1. Retrovirus-related Pol polyprotein from transposon 17.6.
2. Retrovirus-related Pol polyprotein from transposon 297.
3. pol protein [Drosophila melanogaster].
4. pol protein - fruit fly (Drosophila ananassae) transposon Tom (fragment).

It is well known that the phylogenies of transposons *17.6, 297* and *Tom* disagree with the *Drosophila* phylogeny, and they are implicated in horizontal gene transfer [21]. Although a solid prediction of horizontal transfer would require further different analyses, this experiment exemplifies the potential harbored in the association of splits to sequences.

### *Salmonella enterica* Para C

This dataset is of special interest as the contained assemblies from 220 genomes of different serovars within the *Salmonella enterica* Para C lineage include that of an ancient Paratyphi C genome obtained from 800 year old DNA [22, 23], the placement of which is especially difficult due to missing data. As reference, we consider a maximum-likelihood based tree on nonrecombinant SNP data [22, Figure 5a].

**Fig. 4:**
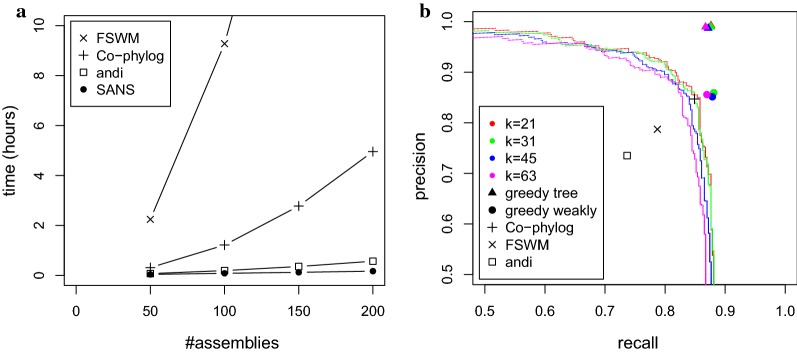
Comparison of running time and accuracy of different methods on the Para C dataset [22] comprising assemblies of $n=220$ genomes. **a** Running time for computing phylogenies on random subsamples. Times for SANS include DBG construction with $k=31$, split extraction and agglomeration. **b** For different values of *k*, weakly compatible subsets (bullets) and trees (triangles) have been greedily extracted. Each point on a line corresponds to a different threshold to discard low weighting splits. Note that both axes begin at 0.5

We studied the running time behavior of the different methods for random subsamples of increasing size. As shown in Fig. 4a, for this high number of closely related genomes, we observed a super-linear running time of up to 41 min for andi, about 5 h for Co-phylog, and up to 43 h for FSWM, whereas the reconstruction of SANS shows a linear increase (Pearson correlation coefficient 0.9994) to about 10 min. The memory requirement of both SANS and Co-phylog remained below 0.5 GB, whereas andi required about 1 GB, and FSWM required up to about 17 GB. We ran CVTree3 with ten values of *k* between 5 and 27, but none of the resulting trees contained more than 5 correct internal edges. For MultiSpaM, we increased the number of sampled quartets from the default of $10^6$ to up to $10^8$, which increased the running time from about 1 h to about 66 h. Both recall and precision improved but were still below 0.2 for internal edges.

The accuracy of the reconstructions with respect to the reference is visualized in Fig. 4b. In particular, we observe: (i) the split reconstruction by SANS and the tree inferred by Co-phylog are comparably accurate and both are more accurate than the FSWM and andi tree, (ii) greedily extracting high weighting splits to filter for a tree selects correct splits while discarding false splits with very high precision, (iii) greedily extracting high weighting splits to filter for a weakly compatible subset also selects correct splits, but, as expected, has a lower precision than the tree filter, because more splits are kept than there are edges in a tree, and (iv) the results of SANS are robust for a wide range of *k* from 21 to 63.

### *Salmonella enterica* subspecies *enterica*

In comparison to the Para C dataset, the 2 964 genomes studied by Zhou et al. [23, 24] are not only a larger but also a more diverse selection of *Salmonella enterica* strains. As reference, we consider a maximum-likelihood based tree on 3 002 concatenated core genes [24, Figure 2A, supertree 3].

**Fig. 5:**
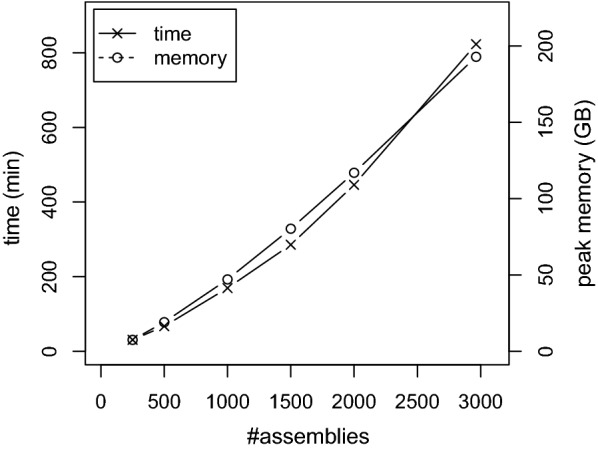
Efficiency on the *Salmonella enterica* dataset [24]. Running time and peak memory usage of SANS include C-DBG construction, split extraction, agglomeration and output. For random subsamples of *n* assemblies, the 10*n* highest weighting splits for $k=21$ have been output. Values have been averaged over processing two random subsamples each

The probability to observe long *k*-mers that are conserved in such a high number of more diverse genomes is lower than for the previous datasets. Hence, we selected a smaller *k*-mer length of $k=21$. To assess the efficiency for increasing number of genomes, we sampled subsets of several sizes. Figure 5 shows the runtime and memory consumption of SANS. In comparison, to process a subsample of size 250 where SANS took about 31 min, andi took about 110 min, whereas Co-phylog and FSWM took already more than 9 h and 50 h, respectively, and MultiSpam was not able to process the dataset at all. We ran CVTree3 with all values of *k* between 6 and 14, but in the best case ($k=8$), the resulting tree contained only 33 (of 247) correct internal edges such that we did not further consider CVTree3 in our evaluation.

**Fig. 6:**
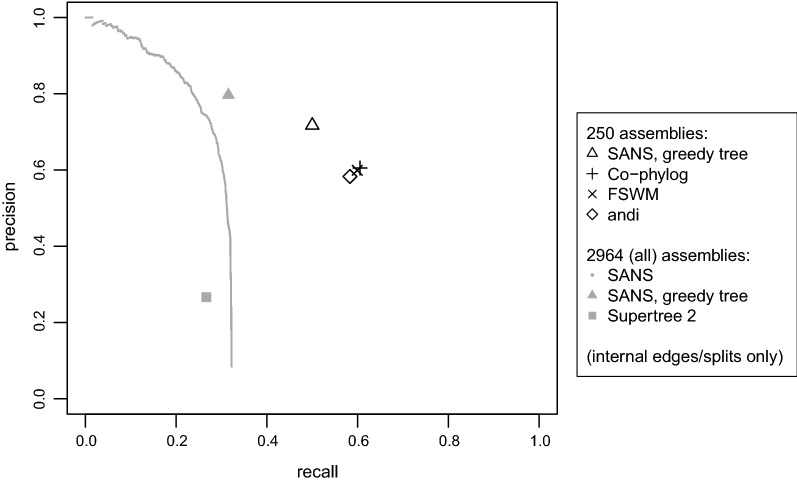
Accuracy on the *Salmonella enterica* dataset with respect to the reference phylogeny [24, Fig. 2A]. Values for subsamples of 250 assemblies have been averaged over two random subsamples. On the gray line, each point corresponds to a different threshold to discard low weighting splits.

The accuracy of the different reconstructions for 250 genomes as well as SANS accuracy on the complete dataset with respect to the reference is visualized in Fig. 6. Co-phylog, FSWM and andi show very similar accuracy for 250 genomes, whereas SANS has slightly lower recall but higher precision (black symbols). For the complete dataset (visualized in gray), SANS proves comparably high precision but low recall. As can be seen by the gray line, the low recall is not caused by filtering all SANS splits for a tree: each point on the line corresponds to a different threshold to discard low weighting splits, and even considering lowest weighting splits does not increase recall.

On the one hand, SANS is rather conservative for large datasets, because any split separating *s* from $n\!-\!s$ genomes requires not only some sequence(s) unique to the *s* genomes but also sequences that are unique to exactly all other $n\!-\!s$ genomes. On the other hand, measuring accuracy by counting correct and false splits corresponding to the topological Robinson-Foulds distance has to be interpreted with care. E.g., a single misplaced leaf breaks all splits between its correct and predicted location. To provide a reference point, the figure also includes the accuracy of another reconstruction proposed in the original study of the dataset, which has been inferred by reconciling the trees of 3002 core genes [24, Figure 2A, supertree 2]. In a quartet-based comparison, this tree agrees to the reference by about 75 percent of all internal edges, whereas, in contrast, less than 30 percent of all internal edges are correct in our Robinson-Foulds measure. A quartet-based evaluation, however, is not meaningful in our case, because our conservative reconstruction is not a fully resolved tree, and missing edges have a much stronger impact on the quartet distance than wrong edges.

### *Ebolavirus*

Viral genomes are short and highly diverse—posing the limits of phylogenetic reconstruction based on sequence conservation. Here we consider 158 complete genomes from five *Ebolavirus* species and two genomes from the outgroup *Marburg* (19 Kbp on average) obtained from the UCSC Ebola Genome Portal [25].

**Fig. 7:**
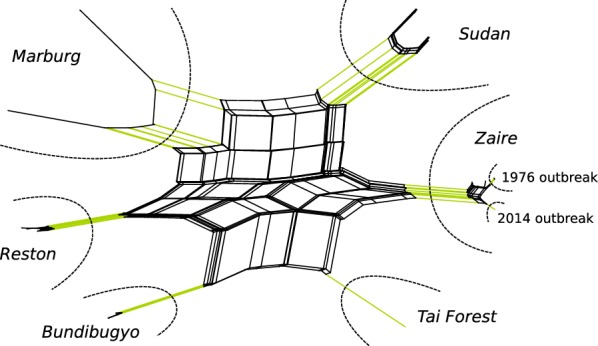
Splits reconstructed for the *Ebolavirus* dataset [25]. A greedily extracted weakly compatible subset of splits obtained by SANS with *k*-mer length 9 have been visualized with SplitsTree [17, 18]. Dashed lines mark different clades: the five *Ebolavirus* species, samples from two *Zaire ebolavirus* outbreaks, and the outgroup *Marburg*. Corresponding splits are highlighted in green

Even with small *k*-mer lengths, the overall sensitivity was too low to infer a fully resolved tree. But the visualization of the inferred splits in Fig. 7 exemplifies the explanatory power of the split framework. While a large fraction of the phylogeny—in particular the inner part—does not show a tree structure, relevant clades are clearly visible: each of the five *Ebolavirus* species as well as the outgroup *Marburg* are well separated, and even samples from two *Zaire ebolavirus* outbreaks build one subclade each.

### *Vibrio cholerae*

The dataset comprises 22 genomes from the species *Vibrio cholerae*, 7 of which have been sequenced from clinical samples and are labeled “pandemic genome” (PG), and the remaining 15 have been sequenced from non-clinical samples and are labeled “environmental genome” (EG) [26, primary dataset]. As already observed in the original study, for these genomes, it is difficult to reconstruct a reliable, fully resolved tree. Nevertheless, representing the phylogeny in form of splits shows a strong separation of the pandemic from the environmental group. The phylogeny presented by the authors of the original study [26, Supplementary Figure 1a] is based on 126 099 sites extracted from alignment blocks.

**Fig. 8:**
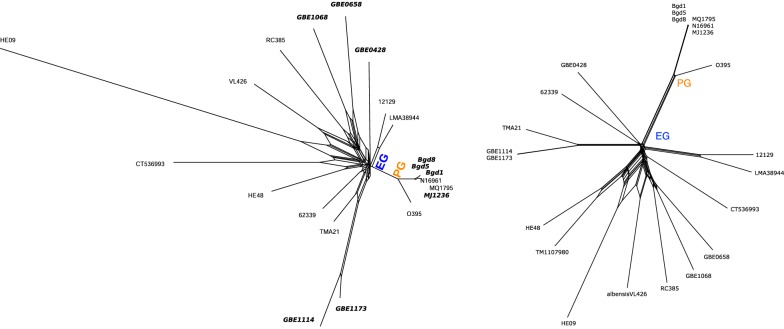
Splits reconstructed for the *V. cholerae* dataset [26]. **a** Greedily extracted weakly compatible subset of splits obtained by SANS visualized with SplitsTree [17, 18]. For taxa highlighted in bold, only read data was available on NCBI (input option -s of Bifrost has been used); for Taxon TM1107980, no data was available on NCBI (February 2019). **b** Reference phylogeny. Figure reprinted from Shapiro et al. [26, Supplementary Figure 1a]

Comparing our reconstruction results to the reference, both shown in Fig. 8, we make two observations. (i) Our reconstruction also separates the pandemic from the environmental group, and agrees to the reference in further sub-groups. (ii) When collecting the sequence data, for some of the genomes, we found assemblies, whereas for others, only read data was available. Because the used C-DBG implementation Bifrost supports a combination of both types as input, we were able to reconstruct a joint phylogeny without extra effort or obvious bias in the result.

## Conclusions

We proposed a new *k*-mer based method for phylogenetic inference that neither relies on alignments to a reference sequence nor on pairwise or multiple alignments to infer markers. Prevailing whole-genome approaches perform pairwise comparisons to determine a quadratic number of distances to finally infer a linear number of tree edges. In contrast, in our approach, the length of conserved sequences is extracted from a colored de Bruijn graph to first infer signals for phylogenetic sub-groups. These signals are then combined with a symmetry assumption to weighted phylogenetic splits. Evaluations on several real datasets have proven comparable or better efficiency and accuracy compared to other whole-genome approaches. Our results indicate robustness in terms of *k*-mer length, as well as the taxonomic order, size and number of the genomes. The analysis of a dataset composed of both assembly and read data indicated also robustness in this regard—an important characteristic, which we want to investigate further.

A distinctive feature of the proposed methodology is the direct association of a phylogenetic split to the conserved subsequences it has been derived from, which is not possible for distance-based methods. For the experiment in "Drosophila" section, corresponding sequences have been extracted manually. We plan to enrich our implementation with a functionality to allow the analysis of characteristic subsequences of identified subgroups, or subsequences inducing phylogenetic splits off the main tree, e.g. horizontal gene transfer.

Another direction of future work is the incorporation of the topology of the de Bruijn graph. Currently, it is simply used as a collection of unitigs. But specific substructures, in particular with regard to the colors in the graph, could be used to identify phylogenetic events.

Finally, we want to emphasize the simplicity of the new approach as presented here. At its current state, apart from iterating a colored de Bruijn graph and agglomerating unitig lengths, the only elaborate ingredient so far is the symmetry assumption realized by applying the geometric mean. We believe that the general approach still harbors much potential to be further refined by, e.g., statistical models, advanced data structures, pre- or postprocessing, to further increase its accuracy and efficiency.

## Data Availability

Source code and test data are available from https://gitlab.ub.uni-bielefeld.de/gi/sans.

## Abbreviations

C-DBG: Colored de Bruijn graph
PG: Pandemic genome
EG: Environmental genome
